# Designing Cardiovascular Implants Taking in View the Endothelial Basement Membrane

**DOI:** 10.3390/ijms222313120

**Published:** 2021-12-04

**Authors:** Skadi Lau, Manfred Gossen, Andreas Lendlein

**Affiliations:** 1 Institute of Active Polymers and Berlin-Brandenburg Center for Regenerative Therapies, Helmholtz-Zentrum Hereon, Kantstraße 55, 14513 Teltow, Germany; skadi.lau@t-online.de (S.L.); manfred.gossen@charite.de (M.G.); 2 Institute of Chemistry, University of Potsdam, Karl-Liebknecht-Straße 25, 14476 Potsdam, Germany

**Keywords:** endothelial cells, bioinstructive implants, vascular grafts, tissue engineering, bioprinting, bioinspired materials, biological membrane, endothelial basement membrane, biomaterial

## Abstract

Insufficient endothelialization of cardiovascular grafts is a major hurdle in vascular surgery and regenerative medicine, bearing a risk for early graft thrombosis. Neither of the numerous strategies pursued to solve these problems were conclusive. Endothelialization is regulated by the endothelial basement membrane (EBM), a highly specialized part of the vascular extracellular matrix. Thus, a detailed understanding of the structure–function interrelations of the EBM components is fundamental for designing biomimetic materials aiming to mimic EBM functions. In this review, a detailed description of the structure and functions of the EBM are provided, including the luminal and abluminal interactions with adjacent cell types, such as vascular smooth muscle cells. Moreover, in vivo as well as in vitro strategies to build or renew EBM are summarized and critically discussed. The spectrum of methods includes vessel decellularization and implant biofunctionalization strategies as well as tissue engineering-based approaches and bioprinting. Finally, the limitations of these methods are highlighted, and future directions are suggested to help improve future design strategies for EBM-inspired materials in the cardiovascular field.

## 1. Introduction

Blood vessels providing the body with nutrients and oxygen are indispensable for human survival. Consequently, malfunctioning blood vessels are associated with cardiovascular diseases (CVD), such as atherosclerosis, myocardial infarction, or stroke [1]. Advanced stages of CVD require interventional therapy, such as vessel dilation by balloons or stent implantation, or vascular surgery to re-canalize, replace, or bypass occluded vessels using vascular grafts [2]. Artificial vascular grafts composed of poly(ethylene terephthalate) (PET) or expanded poly(tetrafluoroethylene) (ePTFE), though still the best alternative after autologous blood vessels, bear a high risk of thrombosis due to their limited endothelialization capacity [3]. Endothelial cells (EC) make up the inner layer of native blood vessels, the tunica intima, forming the endothelium as a functional entity, often referred to as an organ by itself [4]. Its function as a restrictive barrier for small molecules also controls transmigration of blood cells at infectious events, regulates blood cholesterol levels by the uptake of oxidized low density lipoprotein, and determines vascular tone by interacting with underlying smooth muscle cells (SMC) in the tunica media. The primary function of the quiescent endothelium under healthy conditions is the maintenance of an undisturbed blood flow and control of hemostasis. The regulation of this complex interplay ensures a constant and ubiquitous provision of tissues with oxygen and nutrients. The seeding of EC on polymer-based grafts prior to implantation, therefore, suggests itself as an approach to prevent thrombus formation in vivo and to facilitate biological integration of the graft. 

**Figure 1:**
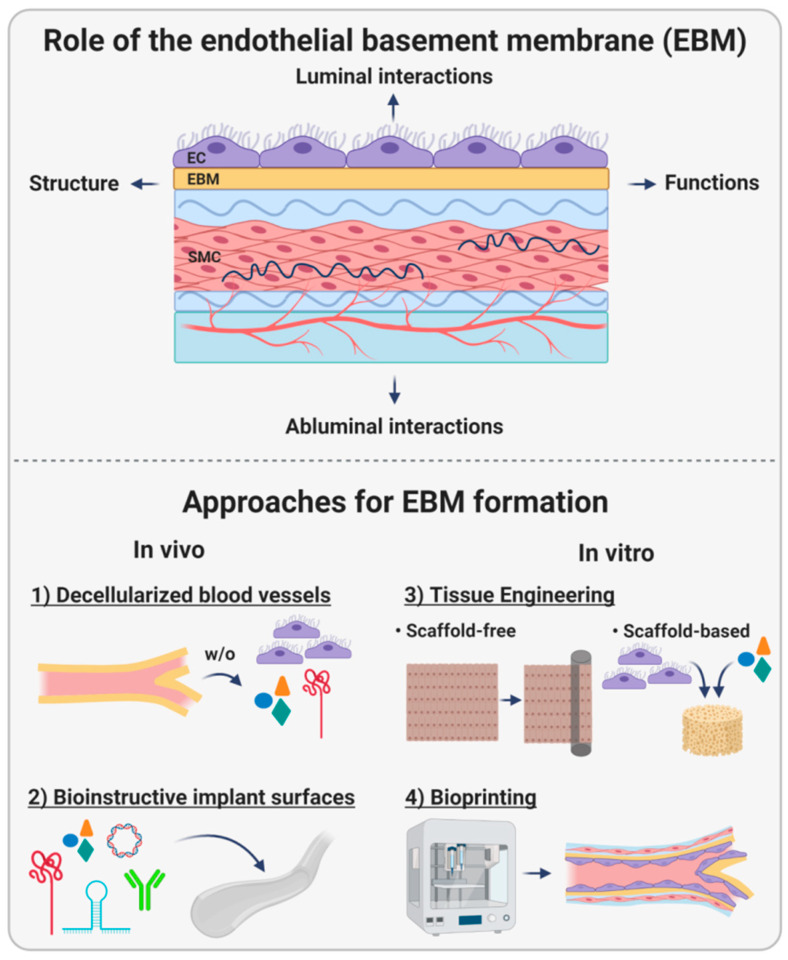
The endothelial basement membrane (EBM). This review describes the role of the EBM, including its structure and functions, especially its luminal and abluminal interactions with adjacent cells. Moreover, multiple design strategies for cardiovascular implants aiming to imitate the EBM or stimulate its formation are described, including decellularization of natural blood vessels, implant biofunctionalization, tissue engineering, and bioprinting.

Endothelialization, including EC proliferation and migration, is a process regulated by several factors and structures, in particular, by the endothelial basement membrane (EBM). This unique structural unit is located underneath every endothelial cell layer, separating it from the underlying tissue. The synthesis and deposition of the EBM are necessary for the firm adhesion of EC to a substrate, and cellular adhesion is a prerequisite for the execution of several EC-related tasks, such as the maintenance of hemostasis. Thus, a thorough understanding of the molecular architecture, its biophysics, and the corresponding functions of the EBM are of utmost importance for more effective implant design strategies, including the creation of new biomimetic implants. Therefore, these elements deserve more focused attention and reflection than currently provided in the secondary literature. This review aims at explaining the importance of the EBM, including its structure, functions, and interactions with the luminal and abluminal sides in vivo. Moreover, strategies to mimic this complex tissue compartment based on decellularization, bioinstructive implant interfaces, tissue engineering, and bioprinting are summarized and critically discussed prior to suggesting future directions to push forward this scientific field (Figure 1).

## 2. The Endothelial Basement Membrane

### 2.1. EBM Composition and Biophysical Properties

Every artery consists of three main layers: tunica adventitia, tunica media, and tunica intima. The outer layer, the tunica adventitia, is mainly composed of collagenous fibers and fibroblasts. In addition, it is pervaded by the vasa vasorum, a network of small capillaries providing the artery with nutrients and oxygen, and the nervi vasorum, a network of nerves controlling vasomotor tone. In large arteries, these networks reach the outer part of the middle layer, the tunica media, which is composed of elastic fibers and SMC and is responsible for the adaption to varying blood pressures by contraction and relaxation. The inner layer, the tunica intima, is made of a thin nonproliferating (quiescent) monolayer of squamous EC forming the endothelium, which primarily enables an undisturbed blood flow. Neighboring layers are separated by elastic laminae: the outer layer is separated from the middle layer by the external elastic lamina, and the inner layer is separated from the middle layer by a complex of the internal elastic lamina and the EBM. The EBM is composed of two basal laminae, each of which consists of a 30 to 40 nm thick electron-lucent lamina lucida/rara adjacent to the cell membrane and a 50 to 80 nm thick outer electron-dense lamina densa [5]. The lamina reticularis, which is not part of the basal lamina but located directly underneath, connects the basal lamina with adjacent connective tissue forming the EBM. While the basal lamina is an extremely fine structural feature of the EBM detectable by electron microscopy only, the complete EBM forms a structure thick enough to be detected by light microscopy. 

**Figure 2:**
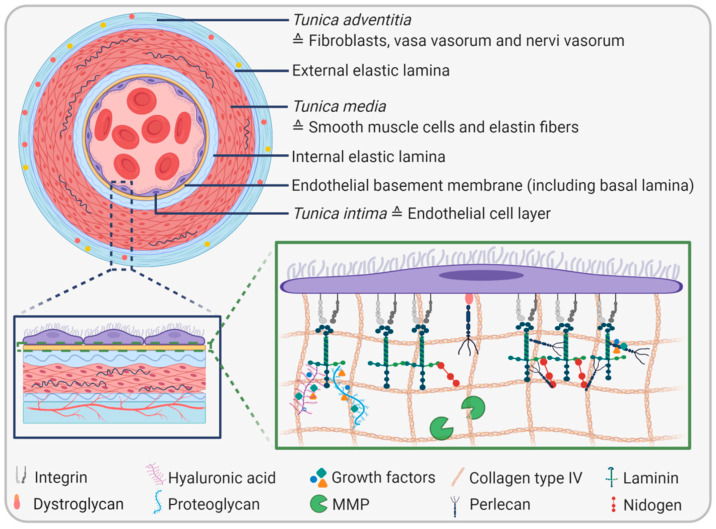
Structure of the endothelial basement membrane (EBM). The EBM (yellow layer) is located underneath the endothelial cell monolayer. The four major components are collagen type IV, laminin, perlecan, and nidogen. These molecules are connected to endothelial cells via adaptor proteins such as integrins and dystroglycans. Moreover, the EBM consists of proteoglycans and glycosaminoglycans, which act as binding partners for numerous growth factors. Proteases such as matrix metalloproteinases (MMPs) are also embedded in the EBM.

The EBM represents a highly specialized part of the vascular extracellular matrix with a thickness of 20–200 nm depending on location in the body, the developmental state, and (patho)physiological conditions [6,7]. The same applies to the composition and structure of the EBM, which is highly heterogeneous and tissue-specific. However, all EBMs share four main components: collagen type IV, laminin, heparin sulfate proteoglycans perlecan or agrin, and nidogens/entactins (Figure 2). Apart from that, additional molecules such as fibronectin, fibulin 1 and 2, collagen type XVIII, and secreted protein acidic and rich in cysteine (SPARC), among others, can occur in the EBM of different vascular beds in a dynamic fashion, but to a lesser extent than the four main components [7]. A systematic proteomic approach identified 202 proteins in the basement membrane of the eye and 144 proteins in the glomerular basement membrane of the kidney, revealing the complexity of this structural unit and its unique tissue-specific composition [8]. Both quantitative and qualitative variations in EBM composition can lead to vascular disorders. For example, an increased thickness of the glomerular basement membrane due to an augmented production of specific basement membrane components is suggested to be a possible biomarker for prediabetes [9].

Collagen type IV and laminin, which exist in different variations, self-assemble into independent sheet-like networks and confer primarily structural support to EC. The remaining main components–heparin sulfate proteoglycans and nidogens–connect the two networks of collagen type IV and laminin with each other [10].

Collagen type IV is nonfibrillar collagen composed of six α chains that assemble into triple helices. Of the three main types of collagen type IV, the most ubiquitous version is the α1α1α2 trimer [11]. Whereas collagen type IV is mainly responsible for the EBM’s structural integrity and, to a lesser extent, for cell adhesion and migration, laminins are the predominant biologically active components. Vascular EC expresses mainly laminin 411 and 511 (isotypes 8 and 10), which are composed of laminin α4 or α5 chains that assemble with β1 and γ1 into complete heterotrimers. The α chain expression depends on the vascular bed, developmental state of the vessel, and the activation state of EC. Laminins bind a number of growth factors such as vascular endothelial growth factor (VEGF), fibroblast growth factor (FGF), and epidermal growth factor (EGF) through several different partners such as heparin-binding domains, hyaluronic acid, chondroitin sulfate, and heparan sulfate. Moreover, they are involved in cell survival, migration, proliferation, and differentiation [10]. Recently, it was shown that loss of laminin 511 is associated with reduced cortical stiffness, smaller focal adhesions, and reduced association with actin–myosin II under flow. Thus, laminin 511 is essential for the shear stress response [12]. Moreover, laminin 511 was shown to contribute to endothelial junctional tightness and thus to the restriction of leucocyte transmigration [13]. Chemical modification of murine laminin 111 by hypochlorous acid or peroxynitrous acid, two potent oxidizing and chlorinating agents formed at sites of inflammation in vivo, leads to compromised cell–matrix interactions, which may, in turn, contribute to EC dysfunction and progression of atherosclerosis [14,15].

Incorporated proteoglycans and associated glycosaminoglycans confer a particularly strong negative charge to the EBM, leading to matrix hydration. Moreover, charge, in addition to size, strongly determines molecule transport across the EBM [16]. Perlecan is composed of a highly conserved core protein that consists of five different domains and binds to three pearl-like strings of glycosaminoglycans, particularly to heparan sulfate, forming the heparan sulfate proteoglycan. It is synthesized by EC and SMC, cross-links basement membrane components, and contributes to the endothelial barrier function. Moreover, it controls vascular hemostasis by limiting SMC proliferation and stimulating EC growth during regeneration [17]. In terms of angiogenesis, it plays a dual role as it has a pro-angiogenic N-terminal domain and an anti-angiogenic C-terminal domain. The pro-angiogenic domain functions as a reservoir for growth factors like FGF-2 and ensures their correct three-dimensional presentation to their respective receptors for the initiation of vessel sprouting. In contrast, the anti-angiogenic action, which is mediated by α2β1 integrin, VEGF receptor 2, and endorepellin, leads to the disassembly of cytoskeletal actin filaments and the termination of cell migration and angiogenesis [18].

Agrin is another type of heparan sulfate proteoglycan and is mainly located in blood vessels in the brain. It stabilizes adherence junctions mediated by vascular endothelial (VE)-cadherin, β-catenin, and zonula occludens-1(ZO-1) necessary for the formation of a particularly tight blood-brain-barrier [19]. However, other data showed that agrin promoted monolayer formation of brain-derived microvascular EC, which lacked proper barrier function as evidenced by a low transendothelial electrical resistance in vitro [20].

Nidogen, also known as entactin, occurs in two different types (nidogen-1 and 2 resp. entactin-1 and 2), consists of three domains, and forms a tight complex with the γ1 chain of laminin. Nidogen-1 is present in nearly all basement membranes, but nidogen-2 occurs predominantly in vascular tissues [21]. Nidogen separately binds collagen type IV, laminin, and perlecan and thus initiates the assembly of a ternary complex of these molecules. Moreover, it mediates cell attachment through its RGD sequence and tethers growth factors, particularly FGF-8 [22].

Aside from growth factors, the EBM sequesters also a variety of proteolytic enzymes, in either soluble or solid states, for remodeling processes. The most prominent proteases are zinc-dependent matrix metalloproteases (MMPs), a protein family of 28 different members in humans, whereas 14 members occur in the vascular system [23]. Especially MMP-2 and MMP-9 were shown to degrade EBM components under both physiological conditions and during cancer progression [24]. Generally, the degradation process is amplified by the resulting matrix degradation products, which further modulate the remodeling process in a manner of a positive feedback loop [10]. Apart from MMPs, other proteases like disintegrin and metalloprotease with thrombospondin motifs (ADAMTS) play a significant role in basement membrane remodeling, mainly in the context of angiogenesis [25].

EC synthesize most of the components of the EBM. However, vascular SMC are also involved in the secretion of specific EBM components, such as laminin 211 [26] and perlecan [27].

The chemical composition alone is insufficient for conducting EBM-associated functions. 

In fact, biophysical parameters such as elasticity, topography, fiber orientation, and anisotropy are equally important to mediate biological actions such as the regulation of mechanotransduction processes [28]. Elasticity results from the composition of the EBM and affects the integrity of the EC monolayer and its associated functions. In principle, a higher basement membrane viscoelasticity leads to increased EC spreading, focal adhesion formation, traction force generation, EC contractility, and disruption of cell–cell junctions [28]. In diabetic mice, stiffening of the basement membrane promotes high glucose-induced retinal endothelial activation [29]. EBM topography, comprising fiber alignment, pore characteristics, and anisotropy, determine EC behavior. For example, substrates with defined grooves and microchannels affect EC alignment, proliferation, and migration [28]. Furthermore, surface patterning and physicochemical variation of silk films synergistically stimulated EC proliferation and differentiation of contractile SMC [30]. 

### 2.2. EBM Functions

In concordance with the heterogeneous structure and composition of the EBM, its functions are similarly diverse. Primarily, the EBM facilitates the firm attachment of EC via integrin-containing focal adhesions and other mediators (for details, see Section 2.3), a prerequisite for the formation of a tight endothelial monolayer with a barrier function. In addition, by virtue of its biochemical composition as well as elastic properties, it promotes and maintains the mature differentiation status of quiescent EC. For example, laminin α5, which is absent in developing EC but present in mature EC, contributes to EC maturation [31]. Moreover, in vitro studies have shown that laminin 411 and laminin 211 drive endothelial differentiation from human pluripotent stem cells [32,33], and matrix stiffness strongly affects EC differentiation; whereas soft substrates promote endothelial differentiation, stiff substrates induce smooth muscle cell differentiation [34]. Apart from integrins, syndecans and dystroglycans also play a role in anchoring EC to the EBM. The syndecan family comprises four members (Syndecan-1, -2, -3, and -4), with EC mainly expressing syndecan-2 and -4. Syndecan-4 was recently shown to link cell-matrix- and cell-cell adhesion. Fibroblasts lacking syndecan-4 produce cell–cell contacts through different cadherins compared to syndecan-4 expressing cells. These are less potent in exerting tension on the ECM, which in turn might lead to reduced cell–cell adhesion [35]. Another study showed a decoupling of vinculin from actin filaments in EC with a reduced expression of syndecan-4. Moreover, syndecan-4-silenced EC exhibited a reorganized actin filament with filopodial protrusions, an altered cell cycle, proliferative potential, and a reduced ability to form tube-like structures [36].

Dystroglycans are the core components of the dystrophin-associated glycoprotein complex, consisting of an α subunit and a β subunit. This complex is present in muscle cells, astrocytes, and EC. Although dystroglycans can interact with different binding partners such as laminin, perlecan, and agrin, laminin is the primary binding ligand. The presence of dystroglycans in EC is clearly associated with angiogenesis [37]. Deletion of dystroglycan leads to impaired retinal arteriogenesis associated with a reduced expression of delta-like ligand-4, implicating that Notch signaling is involved in this process [38]. Galvagni et al. showed that EC interaction with laminin leads to phosphorylation of dystroglycan, which activates the tyrosine-protein kinase Src. This enzyme phosphorylates CD93, a pro-angiogenic transmembrane glycoprotein expressed in EC. This activates the signaling protein Cbl and induces a pathway that leads to EC adhesion, migration, and capillary formation [39].

The firm attachment of EC to the EBM is essential for the formation of an EC mono-layer, which in turn enables an undisturbed blood flow and the prevention of platelet adhesion. This process is also regulated by biophysical parameters such as elasticity and topography, which change in response to shear stress and cyclic stretch. Application of strain, for example, leads to the alignment and densification of collagen fibers, which affect EC functions such as the regulation of hemostasis in a complex and highly regulated manner [28]. 

Another fundamental function of the EBM is the maintenance of a restrictive barrier and the selective filtration of cells, nutrients, growth factors, proteins, hormones, and other molecules from the bloodstream into the connective tissue [40]. Conversely, it is also essential for deporting waste products away from tissues. The underlying mechanisms for the highly selective molecule transport across the EBM are diverse and still not fully understood. It has been a long-held assumption that the transport of molecules across the EBM is mainly size and charge-dependent. Pioneering work by Lieleg et al. has revealed that uncharged molecules are trapped in the matrix, whereas charged particles can pass the electrostatic bandpass filter [41]. However, evidence increases that charge selectivity, especially in the kidney, is nonexistent, suggesting that either other modes of action regulate filter properties or that the regulation is tissue-specific [42]. The EBM’s filter capacity is essential for the maintenance of the blood–brain-barrier protecting the brain from harmful molecules. Lack of the astrocyte-derived laminin α2 chain leads to congenital muscular dystrophy and early death in mice. Likewise, depletion of the γ1 chain results in weakened vascular integrity and hence increased permeability of the blood–brain-barrier. Complete collagen type IV knockout mice are embryonically lethal but also partial knockout of the collagen type IV α1 chain resulted in mice suffering from brain bleeding resembling the pathology of small vessel disease [7]. 

Apart from molecules, the EBM controls the transmigration of leucocytes at events of inflammation. These sites contain less laminin 411, laminin 511, collagen type IV, and nidogen [43]. Moreover, Song et al. found that laminin 511 affects leucocyte extravasation not only directly but also indirectly by modulating the expression of junctional proteins. In particular, laminin 511 activates RhoA signaling through binding to β1 and β3 integrins, resulting in the formation of tight VE-cadherin-mediated junctions between EC. At the same time, the membrane protein CD99L2, which impairs cell-cell interactions, is downregulated by integrin β1 binding, however, not through RhoA signaling [13].

The EBM is also storage of various growth factors and proenzymes and thus a signaling platform for repair, the formation of a provisional tissue, and regeneration, restoring damaged tissue to its normal state. Though repair is thought to be the initial step prior to regeneration, distinguishing the two processes is difficult. Both are regulated by growth factors bound by the EBM. Laminin, for example, can bind numerous growth factors via heparin-binding domains. Incorporating such heparin-binding domains in fibrin matrices improved the retention of VEGF-A165 and platelet-derived growth factor-BB (PDGF-BB) and promoted wound healing in diabetic mice [44]. Besides growth factors, MMPs, which are embedded in the EBM under physiological conditions, play a central role in repair and regeneration. Upon trauma or inflammatory cues, MMPs become activated and degrade their surrounding matrix. The resulting matrix fragments, also called matrikines, initiate further ECM remodeling. This can lead to the activation of immune cells and manipulation of fibroblasts and EC to further stimulate matrix remodeling. In addition, matricellular proteins such as CCN, thrombospondin-1 (TSP-1), or tenascin-C, which are not directly involved in the structural formation of the ECM, serve to tune matrix remodeling in a very specific manner. For example, TSP-1 inhibits angiogenesis by binding to the surface antigen CD36 on activated EC, whereas CCN2 mediates cell migration, proliferation, EBM formation, and vessel stabilization through pericyte recruitment [10].

Finally, the EBM contributes significantly to angiogenesis, either through its structural components and mechanical properties or the embedded growth factors. The role of EBM-derived products in angiogenesis was recently covered in excellent reviews by Arnaoutova et al., Pozzi and Zent, as well as Davis and Senger [45,46,47]. To only highlight a few examples, the γ1 chain of laminin 111 has clearly pro-angiogenic features, whereas the C-terminal domain of the α1 chain of collagen type IV inhibits angiogenesis. Likewise, the α3 chain of collagen type IV prevents FGF-driven vessel sprouting. In contrast, perlecan has both pro and anti-angiogenic properties.

**Figure 3:**
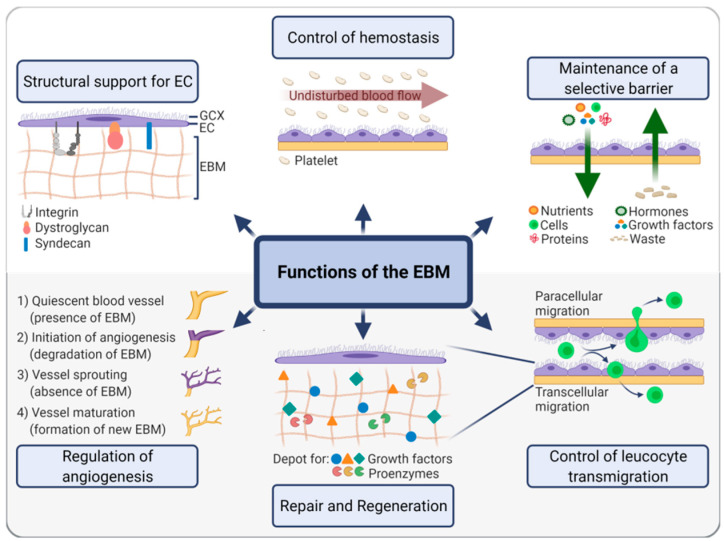
Functions of the endothelial basement membrane (EBM). The EBM confers structural support to endothelial cells (EC) through integrins, dystroglycans, and syndecans. In this context, it indirectly controls hemostasis through EC and thus allows an undisturbed blood flow preventing platelet adhesion. It also functions as a selective barrier for molecules, cells, and waste products. In particular, the migration of leucocytes at events of inflammation is controlled by the EBM. Moreover, the EBM can be considered a depot for numerous growth factors and proenzymes, which are released to induce tissue repair and regeneration. Ultimately, the EBM is a crucial regulator of angiogenesis.

Taken together, the EBM acts towards both the luminal and the abluminal side and evokes a plethora of biological responses (Figure 3). The precise mechanisms of interactions of the EBM at these interfaces are described in the following two sections.

### 2.3. EBM Interactions with the Luminal Side

One of the main functions of the EBM is that of an adhesive substrate for cells. These cell–matrix interactions are predominantly formed by integrin-mediated focal adhesions. Integrins belong to a transmembrane protein family comprising 18 isotypes of α-chains and eight different β-chains. Assembly of one α-chain and one β-chain into heterodimers forms an integrin molecule that, depending on its isotype, binds to different extracellular matrix proteins. EC specifically express integrins binding to collagen (α1β1), laminin (α3β1, α6β1, α6β4), collagen and laminin (α2β1), fibronectin (α4β1 and α5β1), and vitronectin (αvβ3 and αvβ5) [6]. Integrins mediate their cellular responses via their association with numerous actin-binding proteins such as talin, vinculin, α-actinin, paxillin, zyxin, tensin, and filamin. These interaction partners initiate biological responses via different signaling pathways, including those specified by Akt, ERK, JNK, RhoA, Rac1, and Cdc42. Every pathway influences either cell survival, adhesion, migration, proliferation, differentiation, or polarity. These cellular responses are not only dependent on the type of integrin but also on their location and abundance [6]. In addition, there is increasing evidence that cell–matrix interactions also affect cell–cell interactions, suggesting a functional relationship to the barrier function of the endothelium. Since a permeable barrier is associated with the progression and complication of numerous chronic inflammatory diseases, understanding how cell–matrix adhesion influences cell–cell junctions might lead to new opportunities for pharmacological interventions [48].

The transport of cells and molecules from the bloodstream into the connective tissue requires penetration of the endothelial barrier. This can be achieved by a transcellular mechanism through vesicles or by paracellular mechanisms involving cell connecting protein complexes, such as adherens junctions and tight junctions. Endothelial adherens junctions are predominantly composed of vascular endothelial cadherin (VE-cadherin). These transmembrane proteins form calcium-dependent homotypic interactions with extracellular VE-cadherin domains of neighboring cells and anchor to intracellular actin filaments via members of the catenin family. Tight junctions consist of occludin, claudins, and junction adhesion molecules (JAMs). They are linked to zonula occludens proteins inside the cell and further bind to the actin cytoskeleton. The extracellular domains protruding from two adjacent cells form similar homotypic bonds such as adherens junctions. Tight junctions differ from adherens junctions in their reduced resistance to mechanical stress and increased selectivity for particles [49].

**Figure 4:**
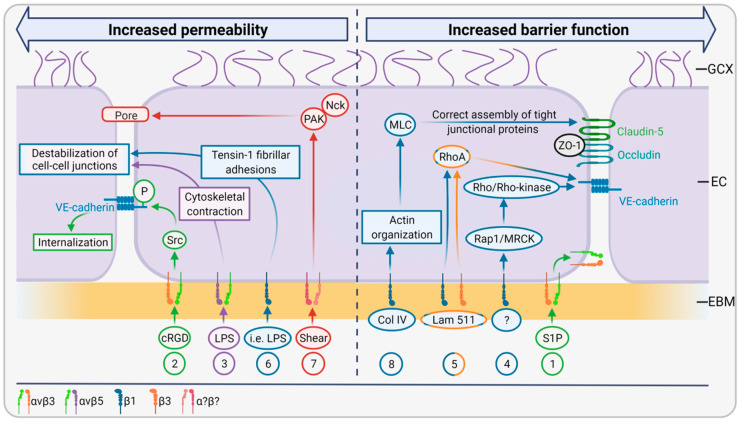
The functional relationship of integrin-mediated cell-matrix and cell-cell interactions. The interactions between the endothelial basement membrane and endothelial cells through different integrins determine the permeability of the endothelial cell monolayer. Here, eight pathways are described; one half are leading to increased permeability and one half to increased barrier function. Integrin αvβ3 can lead to both pathways depending on the stimulus. Whereas sphingosine-1-phosphate (S1P) induces integrin translocation to sites of cell–cell contacts and increases barrier function (1), cyclic arginine-glycine-aspartate (cRGD) activates Src kinase, which phosphorylates VE-cadherin resulting in its internalization and increased permeability (2). Integrin αvβ5 is activated by lipopolysaccharide (LPS), which induces cytoskeletal contractions leading to destabilized cell–cell junctions and thus to increased permeability (3). The integrin subunit β1 can induce both increased permeability and barrier function. Genetic deletion studies have shown that β1 activates Rap1/MRCK and Rho/Rho-kinase, which promote VE-cadherin transport to cell–cell contacts and increase barrier function (4). Similarly, activation of both β1 and β3 stimulates RhoA-induced VE-cadherin localization to cell–cell borders (5). In contrast, β1, when stimulated by inflammatory agents such as LPS, can contribute to the generation of tensin-1 building fibrillary adhesions, which induce endothelial contractility and destabilize cell–cell junctions (6). Pathological shear stress also affects integrin-mediated cell–matrix interactions, whereas the particular subunits remain to be identified. Turbulent shear stress can activate p21-activated kinase (PAK) and its adaptor protein (Nck), resulting in paracellular pore formation and thus increased permeability (7). Finally, β1 can also mediate barrier function via tight junctions. Deletion experiments proved that β1 influences the actin reorganization and phosphorylation of the myosin light chain (MLC), which induces redistribution of the tight junctional proteins claudin-5, occludin, and zonula occludens-1 (ZO-1), resulting in increased barrier function (8).

Generally, the integrin-mediated actions are bidirectionally and can be divided into ‘outside-in’ and ‘inside-out’ signaling. ‘Outside-in’ signaling is initiated by extracellular matrix molecules, which bind to integrin receptors and trigger a signaling cascade inside the cell involving the recruitment and activation of kinases such as Src and FAK. These kinases mediate intracellular integrin clustering and the adhesion of intracellular actin-binding proteins such as talin and kindlin to the cytosolic integrin domain, which leads to a pronounced activation of the ligand-binding function in the extracellular space (‘inside-out signaling‘) [49]. Whereas both pathways are well known in the context of cell survival, migration, and proliferation, their effects on EC barrier function are only beginning to be investigated. Integrin–matrix interactions determine both the transcellular and paracellular permeability of EC. Blockage of the β1 subunit of integrin induces a dramatic transendothelial flux of water and albumin [49]. Moreover, intercellular junctions are weakened in response to changes in integrin-matrix adhesions. Pulous et al. summarized the most recent findings in this field, focusing on VE-cadherin, the key molecule of adherens junctions. Most studies suggest that integrin β3 is essential for barrier function in vivo and in vitro. In mice, deletion of β3 resulted in increased VEGF- and lipopolysaccharide (LPS)-induced leakage in dermal, lung, and intestinal vessels and pretreatment with αvβ3 blocking antibodies prevented hyperpermeability induced by VEGF, transforming growth factor β (TGFβ) and thrombin. In cultured HUVEC treated with sphingosine-1-phosphate (S1P), an angiogenic factor known to increase endothelial barrier function, αvβ3 was shown to translocate to sites of cell–cell contacts and cortical actin [50] (Figure 4 (1)). This phenomenon of integrin localization to sites of cell–cell-junctions was also observed for β1-integrin by Pulous et al., but the significance of this finding remains unclear [51]. In contrast to these studies indicating that β3-integrin promotes barrier function, there is also evidence that activation of β3-integrin by cyclic arginine-glycine-aspartate (cRGD) peptides enhances permeability, most likely by phosphorylation and subsequent internalization of VE-cadherin [52] (Figure 4 (2)). While αvβ5 integrin also seems to affect EC barrier function, it does so opposite to αvβ3. This became obvious from experiments where antibody-mediated blockage of αvβ5 attenuated LPS-induced endothelial permeability in vitro and from αvβ5 knockouts in mice, which exhibited increased survival compared to wildtype controls. It was hypothesized that αvβ5 integrin stimulation leads to cytoskeletal contraction resulting in destabilized cell-cell junctions and thus in increased permeability (Figure 4 (3)).

Next to β3-integrin, studies indicate an important role of β1-integrin for monolayer integrity. Deletion of this integrin in mice evoked VE-cadherin internalization due to the reduced activity of Rap1/MRCK and Rho/Rho-kinase activity, which normally promote VE-cadherin transport to cell–cell contacts [53] (Figure 4 (4)). Song et al. showed that laminin 511 adhesion to both β1- and β3-integrins mediates RhoA-induced VE-cadherin localization to cell–cell borders and thus increases junctional tightness and prevents leucocyte transmigration [13] (Figure 4 (5)). However, another study showed that antibody-mediated blockage of β1-integrin decreased LPS-induced vascular leakage in mice suffering from endotoxemia. Moreover, in endothelial monolayers, β1-inhibiting antibodies reduced monolayer permeability. Mechanistically, inflammatory agents induced β-integrin translocation to tensin-1, building fibrillar adhesions instead of focal adhesions via talin. Fibrillar adhesions, in turn, induced endothelial contractility and destabilized the endothelial monolayer [54] (Figure 4 (6)). Thus, it is suggested that β1-integrin plays a dual role in quiescent and pathological states.

Pathological shear stress also affects endothelial barrier function. For example, integrin-dependent activation of p21-activated kinase (PAK) by pathological shear stress increases endothelial permeability. This process is associated with the interaction of PAK and the adaptor protein Nck leading to paracellular pore formation and thus to vascular leakage. It remains unclear, though, which particular integrin was stimulated in this study [55] (Figure 4 (7)).

Apart from adherens junctions, Izawa et al. showed that β1-integrin-mediated cell- collagen type IV interference by an anti-β1-antibody induces actin fiber remodeling and phosphorylation of myosin light chain (MLC), mediating the reorganization of tight junction-associated proteins such as claudin-5, occludin, and ZO-1 at EC borders. Vice versa, binding of collagen type IV to β1-integrins leads to proper actin filament organization, non-phosphorylation of MLC, and the correct assembly of tight junctional proteins (Figure 4 (8)). Thus, the β1-integrin-mediated cell-matrix interaction ensures a tight endothelial barrier [56].

### 2.4. EBM Interactions with the Abluminal Side

In large vessels, the EBM borders to the internal elastic lamina that separates the EBM from the tunica media rich in SMC.

**Figure 5:**
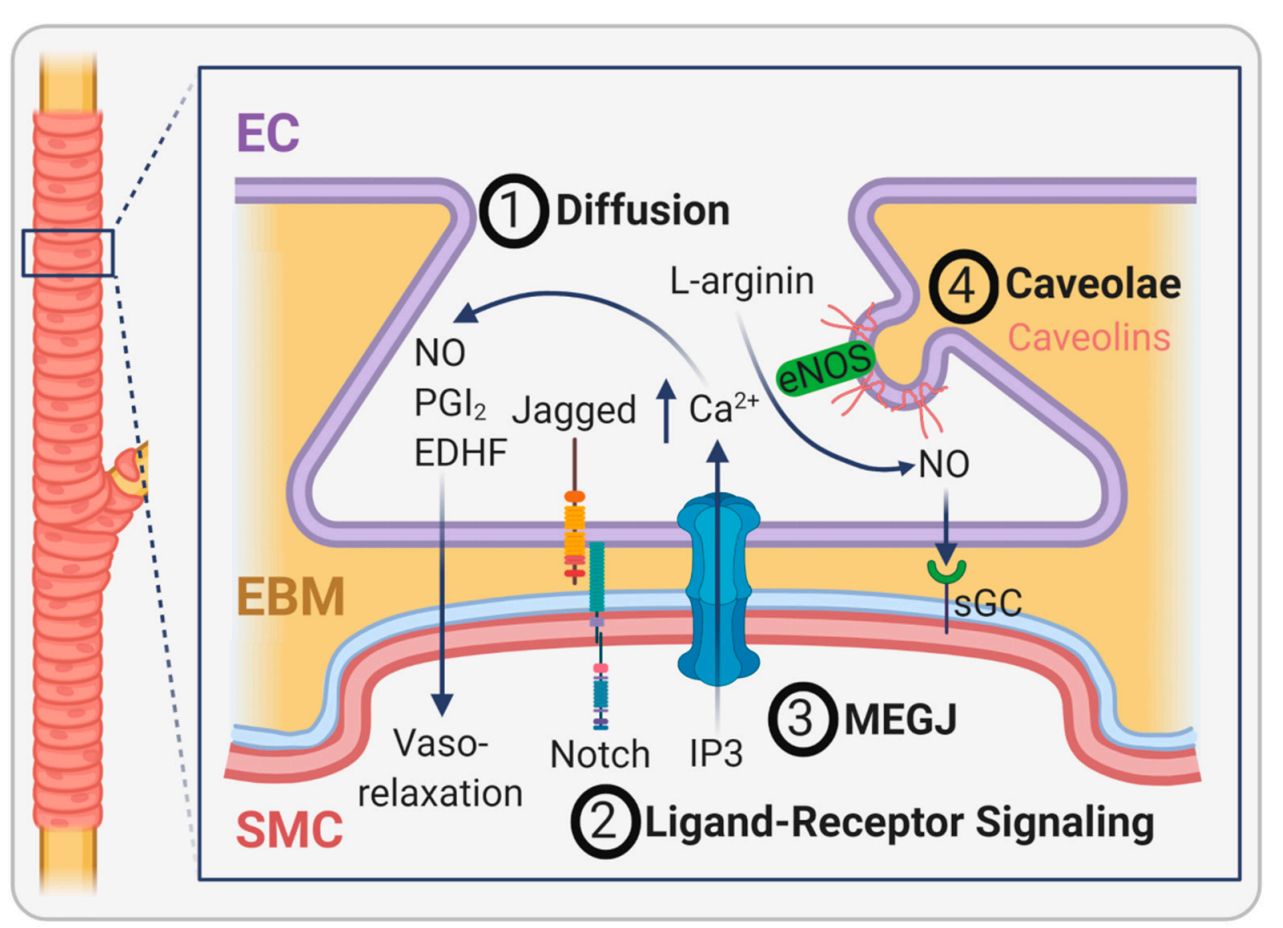
The role of the endothelial basement membrane (EBM) in the interaction of endothelial cells (EC) with vascular smooth muscle cells (SMC). The EBM separates EC from SMC in large vessels. Communication between EC and SMC takes place at myoendothelial junctions through different mechanisms. First, gases like nitric oxide (NO) and small molecules like prostacyclin (PGI_2_) and endothelium-derived hyperpolarizing factor (EDHF) pass the EBM by diffusion. Second, cell membrane-bound ligands (e.g., Jagged) can bind to their respective receptor in the opposing cell (e.g., Notch receptor) to induce a biological response. Third, contact-dependent myoendothelial gap junctions (MEGJ) allow the transport of specific molecules such as inositol trisphosphate (IP3). As a result, the intracellular calcium concentration in EC increases and enhances the diffusion of NO, PGI_2_, and EDHF into SMC, causing vasorelaxation. Fourth, caveolae arrange certain molecules (e.g., endothelial nitric oxide synthase (eNOS)) and its products (e.g., nitric oxide (NO)) in close proximity to its receptor soluble guanylyl cyclase (sGC) to facilitate their interaction.

The functionality of EC and SMC strongly relies on the communication of these two cell types. This takes place at specialized cellular extensions, which can originate mainly from EC but also from SMC and protrude the EBM and the internal elastic lamina through tiny holes towards the opposing cell. These myoendothelial junctions (MEJ), which were discovered more than 60 years ago [57], are signaling microdomains that exhibit a flat or club-shaped phenotype with a width of 0.5 µm and a depth of 0.5 µm [58]. Cell communication at MEJ is achieved either by soluble molecules diffusing between the tunica intima and tunica media or by direct cell–cell contact. The best-described example for EC-SMC interactions via soluble factors is the regulation of vessel tone. Derived from EC, nitric oxide (NO), prostacyclin (PGI_2_), and other hyperpolarizing agents diffuse to SMC and cause vasorelaxation (Figure 5 (1)). Resulting in an opposite physiological effect, EC-derived endothelin and angiotensin II can induce vasoconstriction by SMC [59]. Apart from that, soluble molecules secreted by EC or SMC regulate a high number of other cell responses during development and angiogenesis. A key molecule released by EC is PDGFβ, which binds to the PDGFβ receptor (PDGFRβ) located on SMC and triggers the recruitment of SMC towards developing blood vessels along a concentration gradient. Similarly, EC-released sphingosine-1-phosphate (S1P) is vital for SMC recruitment through G-protein-coupled receptors. SMC secrete angiopoietin-1, a growth factor that binds to the Tie-2 receptor present on EC, stimulating vessel assembly, stability, and cell survival. TGFβ is another growth factor playing a role in EC-SMC interactions, however, in a much more complex manner than the molecules described before. Ultimately, TGFβ determines SMC differentiation, and alterations in the TGFβ signaling pathways are associated with vascular pathogenesis [60]. Only recently, McCallinhart and colleagues showed for the first time that MEJ of mature coronary vessels express numerous types of notch signaling proteins such as Jagged1, Notch1, Notch2, and Notch3, both in vitro and in mice [61]. Since these proteins are well known for their contribution to vasculogenesis, this study reveals that EC-SMC crosstalk via MEJ is essential for blood vessel sprouting (Figure 5 (2)).

Contact-dependent signaling between EC and SMC is predominantly mediated by myoendothelial gap junctions (MEGJ) located at MEJ, which connect the two cell types. Vasculature-specific gap junctions are composed of four connexins (Cx37, Cx40, Cx43, and Cx45), with all but Cx45 found in MEGJ. Although these contact sites between EC and SMC are known for a long time, many open questions regarding their occurrence in different vascular beds and their functionality remain. Some of the reasons are technical hurdles related to the cell preparation methods necessary to obtain transmission electron microscopic images and the lack of a suitable gap junction inhibitor to conduct functionality studies [62]. The primary function of MEGJ is electrical signaling and the transport of second messengers to regulate vessel tone in the adjacent cell. For example, the influx of inositol triphosphate (IP3) from SMC into EC increases intraendothelial calcium levels and activates vasodilatory signaling via nitric oxide and endothelium-derived hyperpolarizing factors (EDHF, Figure 5 (3)). Vice versa, EC can cause SMC hyperpolarization and closure of calcium-dependent voltage channels by EDHF through three different mechanisms leading to vasodilation in small resistance arteries [58]. Regarding the role of specific connexins, Pogoda and Kameritsch found that vasorelaxation is dependent on the connexin composition and post-translational modifications of connexins [63]. Another study showed that MEGJ composed of Cx43 mediates the regulation of angiopoietin-2, which induces vascular hyporeactivity in patients after shock or hypoxia, a state defined as a poor response of vessels to vasoactive substances associated with a high risk for mortality [64].

Apart from gap junctions, the caveolae, which are omega-shaped invaginations in the plasma membrane of many cell types, are found in high density at MEJ. These lipid-rich structures most likely orchestrate numerous signaling proteins such as IP3-Receptor, Cx43, Na+-K+-ATPase, small conductance calcium-activated potassium channels, transient receptor potential cation channel of subfamily V member 4, and endothelial nitric oxide synthase (eNOS), all in close proximity to facilitate their interaction. For example, eNOS is bound to caveolae by caveolin-1, and thus, after conversion of L-arginin into NO, NO is close to its receptor, soluble guanylyl cyclase (sGC), on SMC where it induces vasorelaxation (Figure 5 (4) [58,65].

Taken together, endothelial crosstalk to SMC, which is essential for all vascular functions, requires the penetration of the EBM. With regard to implant materials, numerous strategies were pursued to mimic the EBM in an attempt to improve implant functionality. A selection of these strategies is critically discussed in the following section to reveal their potentials and limitations.

## 3. Strategies to Generate or Mimic Endothelial Basement Membranes

### 3.1. Decellularization of Natural Blood Vessels

Owing to the complex architecture of the EBM, it is preferred to make use of natural EBMs. Decellularization of human and animal-derived tissues became an established approach to obtain mechanically stable matrices consisting of natural ECM components and lacking potentially immunogenic cells. For the fabrication of vascular grafts, a number of different arteries (e.g., umbilical cord, femoral, pulmonary, and internal mammary artery) and veins (e.g., umbilical cord, saphenous, and femoral vein) have been decellularized [66]. To this end, different protocols based on physical, chemical, and enzymatic means were compared with regard to their efficiency in cell removal and integrity of ECM components, as well as mechanical stability of the resulting matrix [67]. For example, decellularization protocols involving increased volumes of detergents and longer incubation times reduced matrix immunogenicity of decellularized equine carotid arteries while burst pressure and suture retention strength remained unaffected [68]. Numerous preclinical studies in animals were performed using pure decellularized grafts or grafts treated with biomolecules such as heparin, growth factors, granulocyte-colony stimulating factor, brain-derived neurotrophic factor, or the matricellular protein CCN1, among others to improve neoendothelialization [69]. 

Despite promising preclinical assessment, only a few decellularized products ultimately entered clinical studies [66]. Among them, decellularized human pulmonary arteries were tested as a patch for cardiac reconstructions (MatrACELL^®^, LifeNet Health, Inc., Virginia Beach, VA, USA). Although many serious adverse events or graft failures were registered, none of these events was device-related. Decellularized human cadaver veins were also tested in hemodialysis patients (SynerGraft^®^, CryoLife Inc., Kennesaw, GA, USA). These grafts did not trigger alloantibody formation in the first three months of the study compared to the control group, indicating reduced immunogenicity of the decellularized grafts. However, immunogenic responses occurred at later time points, revealing the need for further improvements [70]. The combination of biomolecules and growth factors might prevent immune reactions while, at the same time, improve cellular infiltration and vascular wall remodeling. However, such approaches are, for example, associated with the risk of unspecific attraction of platelets and the formation of thrombi. A better solution might come from gene-edited pigs, which lack molecules naturally present in cells from most mammals but are absent in humans and thus provoke graft rejection. Such molecules are, for example, the cell surface carbohydrate α-1,3-galactosyl-galactose (alpha-Gal) and sialic acid N-glycolylneuraminic acid (Neu5Gc) as the terminal part of cell membrane-bound glycans. Since the discovery of the CRISPR/Cas technology, which allows gene manipulation with higher precision compared to conventional methods, progress in this field is advancing rapidly. Fischer et al. engineered pigs with a homogenous knockout of the genes encoding alpha-Gal and Neu5Gc. In addition, three complement inhibitors and two anti-apoptotic and anti-inflammatory genes were overexpressed to prevent endothelial activation and to further reduce the risk of acute vascular rejection. Cells from these transgenic pigs were protected against human complement-mediated cell lysis, and endothelial activation was blocked [71].

### 3.2. Bioinstructive Implant Surfaces for Endothelial Basement Membrane Formation In Vivo

**Figure 6:**
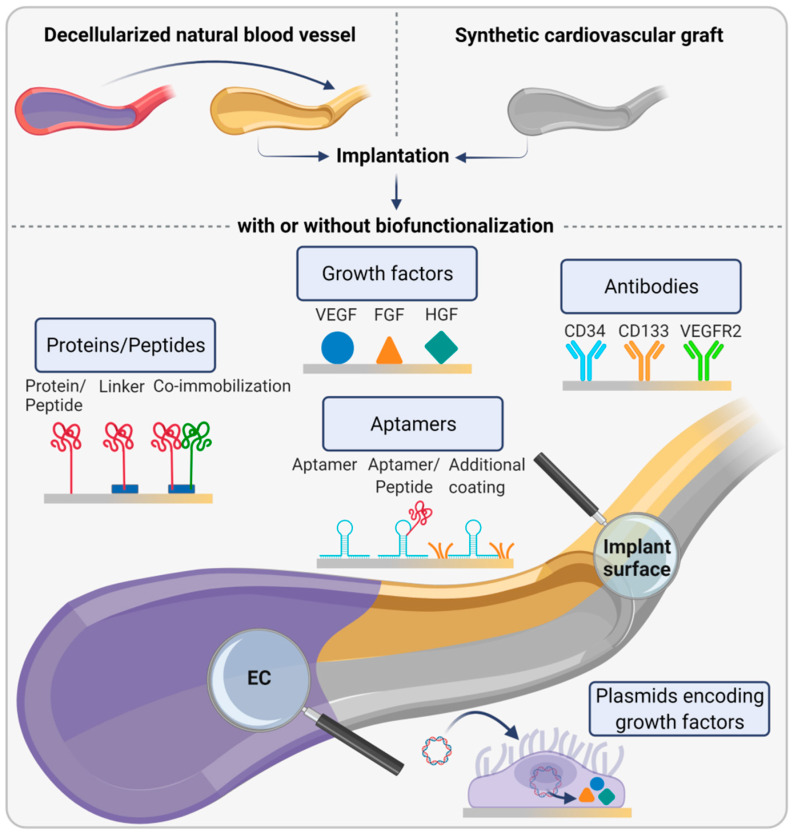
In vivo approaches for recruiting endothelial cells (EC) to implant surfaces. Human- or animal-derived decellularized natural blood vessels or synthetic cardiovascular grafts are used as replacements with or without further modification. Different strategies exist to biofunctionalize implant surfaces in order to promote endothelialization in vivo. The most frequently pursued approach to attract EC is immobilizing proteins or peptides, with or without linker molecules, to implant materials. Another possibility is to coat materials with growth factors such as vascular endothelial growth factor (VEGF), fibroblast growth factor (FGF), or hepatocyte growth factor (HGF) to promote EC adhesion. In a similar approach, plasmids encoding growth factors are attached to implant materials to induce increased local concentrations of these factors after cell transfection leading to enhanced endothelialization. Moreover, aptamers, either alone or in combination with aptamer-bound peptides or surface-bound molecules, are studied for their potential to promote EC adhesion. Functionalization of implant surfaces with antibodies is another approach to specifically capture endothelial progenitor cells and mature EC from the bloodstream.

The use of EC for the generation of an EBM is another plausible strategy bypassing the need to construct such a complex structural unit in vitro. For this purpose, implant surfaces are functionalized with biomolecules to promote EC migration from surrounding vessels and subsequent EBM formation (Figure 6).

One approach to attract EC is precoating implants with ECM molecules or peptides to facilitate EC adhesion. Here, the biggest challenge is to prevent the adhesion of platelets, which exhibit an affinity to proteins similar to that of EC. In terms of stent surface modification, a nanocoating of stromal cell-derived factor-1α and laminin inhibited platelet adhesion and activation while endothelialization was enhanced. In vivo, thrombus formation and neointimal hyperplasia were prevented, suggesting that such ECM-inspired modifications improve stent functionality and longevity [72]. Also, collagen and laminin, constituting integral components of the EBM, were widely used as coating substances. Both proteins increased endothelialization, however, with a reduced potency compared to peptides [73]. The effects of peptide coatings were recently reviewed by Radke et al. [74]. The Arg-Gly-Asp (RGD) peptide, present in fibronectin and responsible for strong cell adhesion, is very efficient in promoting EC adhesion and their migration in vascular grafts [75]. Moreover, the combination of RGD with peptides occurring in the laminin β chain, such as YIGSR, significantly enhances EC proliferation [76]. GFPGER, a protein sequence occurring in collagen type I, increases endothelialization more efficiently than collagen and laminin and even reduces platelet adhesion in an ex vivo arteriovenous shunt model under antiplatelet therapy [73]. From a biomimetic point of view, the use of mussel-inspired proteins such as polydopamine is of interest. In mussels, dopamine enables their firm adhesion to stone under water in the presence of wave shear. Thus, the polydopamine coating has often been used as a linker to enhance peptide adhesion to a substrate. Immobilization of serum albumin and a peptide aptamer for endothelial progenitor cells (EPC) via polydopamine significantly improved EPC adhesion and simultaneously reduced platelet adhesion and fibrinogen adsorption in vitro [77]. Likewise, ePTFE functionalized with polydopamine and coupled to an NO catalyst and VEGF increased NO production, promoted EC proliferation, and prevented platelet adhesion [78]. Coimmobilization of the antithrombotic peptide ACH11 and Cys-Ala-Gly (CAG), a tripeptide present in collagen type IV of the EBM, was shown to have a selective affinity for EC but not for SMC, thus reducing the risk for vessel narrowing due to excessive SMC proliferation (intimal hyperplasia). Moreover, the two peptides enhanced EC adhesion, proliferation as well as the release of NO, and they improved patency rates in vivo [79]. Moreover, high throughput screening identified a cyclic integrin ligand named LXW7, which contains four unnatural D-amino acids rendering it more proteolytically stable than purely natural peptides. This ligand improved EPC attachment, proliferation, and differentiation in vitro and resulted in an extremely high patency rate of 83% after six weeks from implantation in rat carotid arteries compared to 17% patency in control animals [80].

Another strategy involves the attachment of EC-attracting growth factors, mainly VEGF, to the implant surface. VEGF immobilized to polyurethane (PU) via polyethylene glycol as a spacer molecule selectively induced EC adhesion and proliferation without affecting platelet adhesion [81]. Likewise, PEG-coated cerium oxide nanoparticles, which have the potential to inhibit oxidative stress-induced EC apoptosis, and free VEGF embedded in polyurethane scaffolds, facilitated endothelialization [82]. A similar approach is based on the linkage of plasmids encoding growth factors leading to cell transfection and increased local expression of the respective protein. For example, plasmids encoding hepatocyte growth factor (HGF) immobilized on a substrate using polydopamine resulted in the transfection of both EC and SMC. This induced an increased proliferation of EC but not of SMC in vitro, which suggests that HGF is a potential promotor of endothelialization, which also prevents intimal hyperplasia [83]. Moreover, stents coated with VEGF-expressing plasmids resulted in increased local VEGF concentrations in rabbit aortas, most likely due to transfection of EC as shown in vitro, and improved endothelialization, whereas stent stenosis was inhibited [84]. In this context, aptamers, which are short single-strand nucleic acids fragments or aptamer–peptide complexes, were also studied regarding their potential to recruit EC. Recently, Schulz et al. showed that poly(ether imide) films functionalized with an aptamer-cRGD peptide improved the initial adherence and shear resistance of EC compared to unmodified films in vitro [85]. Another study investigated stents coated with heparin and aptamers regarding their potential to capture EPC and found that this modification effectively trapped arrested cells under dynamic conditions and regulated their distribution [86].

A promising technique uses capture antibodies to catch EC and EPC from the bloodstream to hold them in place. The most frequently studied antibodies are directed against specific markers of EPC (e.g., CD34 and CD133) and fully differentiated EC (e.g., CD31 and VEGFR2). For example, coating of stents with VEGFR2 antibody fragments enhanced endothelialization in vitro and in vivo, as shown by increased EC adhesion and the formation of an endothelial monolayer (neointima) after 30 days in porcine coronary arteries [87]. Moreover, surface coating of ePTFE with ECM and monoclonal anti-CD34 antibodies significantly increased EC adhesion and decreased platelet attachment [88].

Despite some promising progress in this field, none of the described methods has successfully passed clinical trials. The use of peptides is sometimes associated with their detachment from the implant surface due to their small molecular weight, which makes them useless for cell capturing. CD34 specific antibodies can evoke intimal hyperplasia at the vessel–implant-interface and adhesion of platelets and proteins, which displays a high risk for thrombosis. Moreover, the long-term patency of antibody-coated implants remains to be determined. Finally, the manufacturing of antibodies is expensive, which further compromises their commercial application [74]. Because of these limitations, research has shifted back to in vitro studies with a particular focus on tissue-engineered and bioprinted EBMs.

### 3.3. Tissue-Engineered Endothelial Basement Membranes

**Figure 7:**
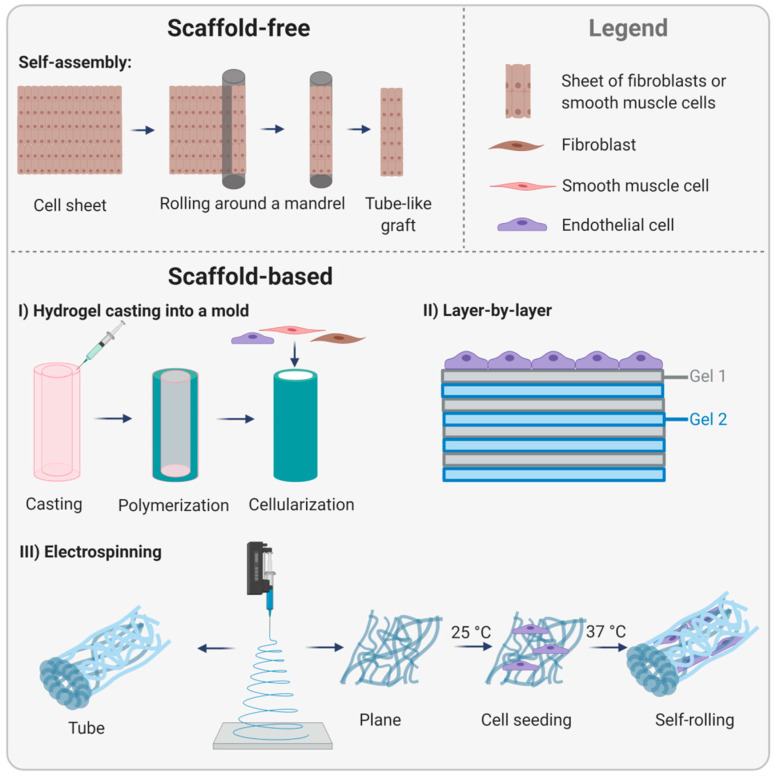
Tissue engineering-based strategies to mimic the endothelial basement membrane (EBM). A scaffold-free method based on cellular self-assembly and three different scaffold-based approaches are shown. The latter comprises hydrogel casting into a mold, hydrogel polymerization, scaffold removal and graft cellularization (I), layer-by-layer stacking of two different hydrogels and EC cultivation on top (II), and electrospinning of either tubular grafts or fabrication of plane sheets with shape-memory polymers, which self-roll into tube-like constructs at a specific temperature (III).

Based on the hypothesis that artificial scaffolds interfere with the natural organization of cells and prevent physiological tissue formation, scaffold-free methods became a key research topic. Pioneering work was conducted by L’Heureux et al. in 1998, who invented the self-assembly strategy. For this, fibroblasts were cultivated in conventional culture flasks and successively rolled around a cylindrically shaped mandrel to form a tube-like construct (Figure 7, upper part). To mimic the cell-free internal elastic lamina and the EBM serving as a barrier for SMC migration into the lumen and as a substrate for EC, the construct was air-dried to induce cell death. In the next step, a sheet of SMC was wrapped around the construct to mimic the tunica media. Then, a fibroblast-containing sheet was rolled around the SMC layers to generate the tunica adventitia. Finally, the luminal area was seeded with EC. Maturation of these grafts in a bioreactor under dynamic conditions increased the stability reaching burst pressures comparable to human vessels. However, in dogs, these implants, tested without endothelialization, failed already after only seven days [89]. To reach clinical translation, the complex manufacturing process had to be simplified, and possibly more importantly, cells of the target population, such as cells from elderly patients suffering from cardiovascular diseases, had to be used. In subsequent studies, patient-derived fibroblasts were successfully used as the only cell type to facilitate the process. In addition, the maturation phase under dynamic conditions was prolonged. Having made these modifications, self-assembled vascular grafts were used in first preclinical studies (LifeLine™, Cytograft Tissue Engineering, Inc., Novato, CA, USA). Data showed enhanced burst pressures compared to the first study, and grafts remained patent for eight weeks in a primate model [90]. In 2007, the first clinical study using LifeLine grafts as arteriovenous shunts in the forearm of 10 patients requiring vascular access for hemodialysis was conducted. In this trial, air-dried patient-specific fibroblasts were used to manufacture self-assembled grafts, and autologous EC were seeded on the luminal side seven days prior to implantation. Of ten patients, one patient died due to reasons not associated with the vascular graft, and three grafts failed due to thrombosis, dilation, or aneurysm. The remaining six grafts showed a promising patency rate of 78% after one month and 60% after six months [91]. The major limitation of this type of vascular graft is the extraordinarily long time for preparation and maturation of up to 7.5 months. Thus, further research focused on establishing off-the-shelf vascular graft strategies and foregoing autologous EC. Specifically, allogeneic approaches were made to circumvent the time-consuming process of patient-specific cell isolation and propagation prior to generating a cell-containing graft. In 2014, the allogeneic LifeLine graft, composed of allogeneic fibroblast sheets were frozen at −80 °C until implantation, then thawed and implanted without seeding of autologous EC. This approach did not evoke immunological responses while reaching sufficient mechanical strength that was not affected by the freeze–thaw cycle [92]. Recently, a thorough characterization of cell-assembled ECM and the effect of the devitalization process by dehydration was performed. This study revealed the complex composition of more than 50 different proteins and no changes in the biological architecture and mechanical properties after devitalization [93]. Despite these positive results [94,95], the extremely long production time remained an issue for future clinical use. The latest work by L’Heureux et al. aimed not only at overcoming this hurdle but also extended the application area of cell sheet-derived matrices from vascular grafts to other areas such as sutures. For this, fibroblast-derived sheets were produced according to the conventional protocol, frozen, thawed, and rehydrated prior to cutting the sheet into thin threads that could be used as a yarn. Skin wounds of rats sutured with this yarn were completely closed after 14 days, and histological analysis showed no signs of inflammation. As a proof of concept, a textile-like woven tubular graft was produced that withstood higher burst pressures than internal mammary arteries and exhibited superior suture pullout strengths over synthetic materials. Moreover, short-term implantation in the carotid artery of sheep proved implantability with low transmural permeability only and normal blood flow [96]. Other groups were also able to successfully apply the self-assembly strategy indicating the robustness of this method; however, most studies used vital cell sheets. For example, self-assembled SMC-based grafts that were periodically exposed to high hydrostatic pressure during repeated cell seeding resulted in grafts of high elasticity important for vasoreactivity [97]. Bornstädt et al. modified the technique and used living sheets of fibroblasts and SMC to construct a small diameter vascular graft that was stabilized by a degradable glue during maturation in a bioreactor. The glue significantly shortened the total production time of the graft. Histological analysis after eight weeks of implantation in rats showed that the grafts structurally resembled native arteries [98].

Whereas L’Heureux and other researchers rely on scaffold-free approaches without focusing on the EBM in particular, other lines of research aim at the production of biomimetic materials resembling specifically the EBM. This strategy is based on the hypothesis that scaffolds serve as an orientation point for cells, thus guiding colonization, proliferation, and new tissue formation in a directed manner. Weinberg and Bell first introduced scaffold-based vascular tissue engineering in 1986 with tissue-engineered vascular grafts composed of a collagen gel, bovine fibroblasts, SMC, and EC [99]. Though the mechanical stability was poor, this attempt initiated a plethora of subsequent studies aiming to improve this promising approach. Niklason and colleagues developed polyglycolide-based tubular scaffolds that were cellularized with SMC. To reduce immunogenicity, scaffolds were decellularized without compromising their mechanical properties. These grafts could be stored long-term at 4 °C, and animal studies demonstrated excellent patency and no signs of dilation, calcification, or intimal hyperplasia [100]. Thus, these grafts were considered an option for acute cases and were, until now, transplanted in 240 patients requiring hemodialysis access [101]. Niklason et al. also pursued the manufacturing of vascular grafts made of fibrin gels, a material that has numerous advantages for tissue engineering purposes but lacks mechanical stability. Fibroblasts and SMC were suspended in fibrinogen gels prior to injection into tubular molds. After polymerization of fibrin, the scaffolds were cultivated for 30 days in a bioreactor under pulsatile stretching. Engineered blood vessels demonstrated average burst pressures over 900 mm Hg, which is much higher than results from other groups working with fibrin [102] (Figure 7, lower part, I). For more information on the history of bioengineered blood vessels, the reader is referred to the latest review by Niklason and Lawson [103].

In addition to strategies using natural cell-derived EBMs, attempts to mimic the EBM prior to cell cultivation were made. One example is the layer-by-layer technique. To mimic the corneal EBM, ultrathin layers of collagen type IV and laminin were placed on top of a collagen type I film. Bovine corneal EC cultured on top of these artificial EBMs formed denser monolayers than the control and exhibited a more pronounced expression of the tight junction protein ZO-1 and cortical F-actin filaments showing that these constructs displayed a selective barrier preventing cell migration but allowing cell-cell communication [104]. There is evidence that the fabrication protocol can be extended to other components of the EBM to generate tissue-specific basement membranes [105] (Figure 7, lower part, II).

Electrospinning is a versatile method for the production of fibrous scaffolds composed of natural or synthetic polymers mimicking the structure and functions of the ECM, including the EBM. PU is a widely used polymer owing to its biocompatibility and biodegradability. Electrospun PU functionalized with polydopamine and coated with heparin and VEGF inhibited platelet adhesion and enhanced EC proliferation in vitro. Thus, this type of scaffold modification is potentially promising for the development of hemocompatible vascular grafts [106]. Yu et al. studied the effect of fiber thickness on the endothelial cell response. For this, polycaprolactone membranes with different fiber diameters were electrospun and seeded with porcine EC. Fine fibers of 54 nm average diameter approached the biofunctionality of the EBM more efficiently than thicker fibers, both with regard to cell adhesion and proliferation. Moreover, additional coating with collagen type IV enhanced not only cell proliferation and viability but also the elastic modulus and the adhesion force [107]. Apart from the scaffolds composed of a single material, hybrid scaffolds composed of two or more materials might exhibit more positive effects due to synergy than the materials alone. In this context, hybrid scaffolds of electrospun methacrylated gelatin (GelMA) and poly(ε-caprolactone) (PCL) were tested in different ratios to identify the ideal composition for mechanical stability and biological features necessary for EC function. Data showed that a ratio of 1:1 met the mechanical requirements of vascular grafts and supported EC adhesion and the expression of molecules essential for the formation of a confluent monolayer [108]. Subsequently, hybrid scaffolds are combined with shape-memory materials able to change their shape in response to external stimuli. This approach is of particular interest for endothelialization strategies aiming to overcome the difficulty of seeding EC uniformly onto the luminal side of a tubular vessel construct.

In a study by Zhao et al., flat scaffolds of the shape-memory polymer poly[lactide-*co*-(trimethylene carbonate)] (PLATMC), which are initially tube-shaped, were covered with an electrospun nanofibrous membrane of poly(ε-caprolactone) and methacrylated gelatin and conveniently seeded with EC. These scaffolds were cultivated for 24 h at 25 °C to allow cell attachment. Thereafter, they were cultivated at 37 °C, which induced PLATMC to self-roll into tubular constructs. Immuno-staining showed a uniform endothelial cell layer expressing VE-cadherin and vinculin after three days of culture, indicating proper cell–cell and cell–matrix contacts [109]. In a similar approach, SMC were guided by temperature-responsive shape-memory scaffolds to align circumferentially. The shape-memory polymer poly[lactide-*co*-glycolide-*co*-(trimethylene carbonate)] (PLGATMC) was used in combination with electrospun membranes of aligned fibers of poly(lactide-co-glycolide)/chitosan (PLGA/CS) to promote cell adhesion and proliferation. After SMC seeding and attachment onto planar scaffolds at room temperature, scaffolds were placed at 37 °C to induce self-rolling. Scaffolds with a membrane of PLGA/CS of a ratio of 7:3 displayed best cell proliferation [110] (Figure 7, lower part, III).

### 3.4. Bioprinting Endothelial Basement Membranes

**Figure 8:**
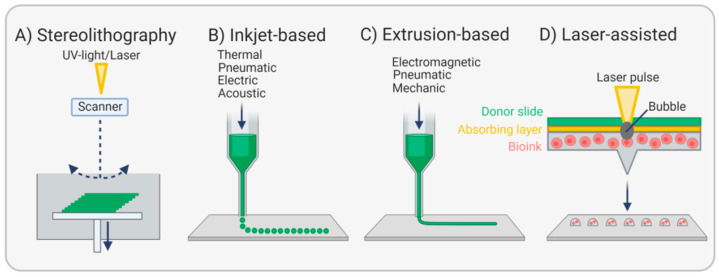
Bioprinting of constructs leading to endothelial basement membrane formation (EBM). Stereolithography is based on photopolymerizable bioinks that polymerize in a layer-by-layer manner in response to light energy (**A**). Inkjet-based bioprinting is a nozzle-based technique printing small drops of bioink (**B**). Extrusion-based printing is also nozzle-based but produces bioink fibers instead of drops. Both Inkjet- and extrusion-based printing can operate with various energy sources (**C**). Laser-assisted bioprinting generates cell-containing hydrogel-based bubbles in a rather gentle procedure (**D**).

Since 1986, when Charles W. Hull set the stage for 3D printing by describing the stepwise solidification of a photopolymer by UV light, a method he called stereolithography [111] (Figure 8A), the progress in this technology is advancing rapidly, and a myriad of studies was undertaken aiming at the fabrication of whole tissues and organs.

Compared with the approaches mentioned above for EBM reconstruction, bioprinting enables precise control over the composition, spatial distribution, and architectural complexity of printed specimens. Moreover, bioactive substances such as growth factors can be accurately embedded to better guide tissue regeneration. Beyond that, the computer-driven automated process facilitates high reproducibility. Nevertheless, the printing of the complex hierarchical structure of a vascular tree, including vessels of varying diameters (arterioles, capillaries, venules), remains a substantial challenge. To accomplish this goal, both direct and indirect printing approaches have been pursued. While direct printing means that bioinks are directly deposited on a substrate, indirect printing takes advantage of temporary support materials. These so-called sacrificial/fugitive materials are printed in cell-laden matrices in the desired structure and removed shortly after that by temperature change or solvents leaving, for example, hollow capillary-like channels in the matrix. In the following step, these channels can be perfused with EC to mimic the endothelium [28]. While numerous studies focused on printing microvasculature for the integration in various types of tissue and organs [112,113,114,115,116], comparably few studies focused on printing EC monolayers and three-layered vascular grafts [117].

Inkjet-based or drop-on-demand bioprinting is ideal for the high-resolution fabrication of small tissues, including EC monolayers using tiny droplets of bioink (Figure 8B) [118]. For example, three-dimensional, neatly layered retina models could be printed layer-by-layer based on gelatin methacrylate to simulate the basement membrane. In a second step, retinal pigment epithelial cells and photoreceptor cells were deposited in a specific micropattern onto the EBM substitute and cultivated for three days in vitro. Both cell types proved to be functional, as shown by the expression of cell-specific markers and the release of VEGF, and could be used to study sight-threatening diseases [119].

Huber et al. applied stereolithography for the fabrication of small diameter tubular structures made from photo-curable polyacrylates. These grafts were branched and porous to allow cell adhesion and nutrient exchange. Endothelialization was supported by material functionalization with heparin and RGDC peptides to mimic the EBM. A rotating seeding procedure achieved a homogeneous endothelial cell lining. However, platelet adhesion was also significantly increased, revealing a potential risk for thrombosis [120]. Extrusion-based printing or direct writing, a method that uses electromagnetic, pneumatic, or mechanical forces to obtain a filamentous bioink structure, was most studied (Figure 8C). For example, triple-coaxial extrusion of three different bioinks resulted in the fabrication of 2-layered vascular grafts resembling the tunica media and tunica intima. A mixture of alginate and decellularized ECM derived from the porcine aorta was used as a bioink and either enriched with SMC for the outer layer or EC for the inner layer. To stabilize the tubular shape during the printing process, the core of the construct was filled with sacrificial poly-(ethylene glycol)-block-poly-(propylene glycol)-block-poly-(ethylene glycol) (PEG-PPG-PEG), which was removed after the production process. Implantation of these grafts in rats for three weeks showed high patency, an intact endothelium, and remodeled smooth muscle [121]. In another study, vascular grafts composed of mainly alginate and bacterial cellulose were extruded in a calcium chloride-containing bath and resulted in highly flexible and robust grafts. These grafts showed sufficient hemocompatibility and lacked cytotoxicity. After implantation in rabbit carotid arteries, grafts remained stable over a period of one month despite the strong encapsulation with connective tissue [122]. Nevertheless, alginate is not ideal for mimicking the EBM of native vessels due to missing cell adhesion sites [117]. In contrast, fibrin is a highly promising natural polymer for numerous vascular tissue engineering applications due to its occurrence in blood, its adhesion sites for multiple cell types, and its degradability, making it attractive for autologous strategies. From a mechanical point of view, however, it is too unstable to build vascular grafts of sufficient strength, and its viscosity is too low for bioprinting. To overcome these limitations, fibrinogen was mixed with dermal fibroblasts and gelatin, a collagen-derived polymer that possesses good printability properties, and extruded onto a polystyrene substrate coated with PEG-PPG-PEG using a rotating bioprinter. After cultivation for two months, increased collagen deposition was observed, and mechanical strength data showed that grafts were half as stable as human saphenous veins. Thus, fibrinogen, in combination with other materials, might eventually become a suitable bioink for vascular grafts [123]. 

Among the printing techniques, laser-assisted bioprinting or laser-induced forward transfer is considered to be particularly cell-caring. The technique is based on a laser pulse directed on a donor slide sitting on an absorber substrate and a cell-laden bioink. The laser pulse generates a bubble, which liberates cells from the bioink and allows the controlled deposition on a substrate underneath (Figure 8D). Though this technique is superior in terms of cell viability and resolution when compared with the three aforementioned methods, further progress in terms of realizing vascular structures is mandatory [113].

## 4. Challenges and Future Directions

Numerous strategies have been pursued to generate biomimetic materials that resemble or stimulate the formation of the natural EBM in order to support endothelialization or to construct complete vascular grafts. However, only a few products entered clinical studies, indicative of the obstacles still to overcome. 

Decellularization of animal-derived vessels still poses a risk of immune response despite the high similarity of animal- and human-derived ECM molecules. Moreover, the removal of those immunogenic compounds is associated with decreased mechanical strength. Human vessels would circumvent this issue, but the availability of such vessels is limited. A potential solution might come from gene-edited pigs genetically engineered to lack molecules that are highly immunogenic for humans. The in vivo recruitment of EC for vascular grafts by different implant biofunctionalization strategies is primarily associated with unwanted platelet attraction and thrombus formation and with the risk of stimulating SMC proliferation, bearing risk for neointimal hyperplasia and subsequent vessel constriction. Bioprinting, the latest among the techniques described here, is still connected with a number of technological hurdles. One of them is the identification of suitable bioinks that exhibit a viscosity low enough to be printed but high enough to generate a stable construct. Another hurdle is nozzle clogging, especially when printing high cell numbers. A potential solution might come from 4D printing, a novel approach that includes the additional parameter of time after the manufacturing process. For this purpose, stimulus-responsive materials such as shape-memory materials are used that change their 3D structure over time after printing upon a specific stimulus. It remains to be investigated whether 4D printed objects are superior to classically printed specimens, owing to their ability to adapt to a dynamic environment [112,124]. 

Among the tissue engineering-based strategies, electrospinning, though a highly versatile tool that mimics ECM properties, is limited in its ability to control pore size, complicating cell infiltration and remodeling processes. Moreover, scaffold degradation and the associated effects of degraded products in the organism remain issues that need to be addressed in long-term studies. In contrast, the self-assembly method by L’Heureux et al. appears most promising in creating vascular grafts despite the extremely time- and cost-intensive process. Referring to their latest work [96] on fibroblast-derived ECM-based yarn used to fabricate a vascular graft of extremely high mechanical stability, future studies will answer questions concerning the ability to promote in-situ endothelialization, growth, and remodeling, as well as compliance in response to blood pressure changes. In this context, the reader is referred to the latest review by Zilla et al., one of the pioneers in cardiovascular tissue engineering from the perspective of a cardiothoracic surgeon [125]. In this article, half a century of research in this field is critically, if not pleadingly, discussed trying to understand why a proper implantable graft is still lacking. A central issue to work on, according to Zilla et al., is the understanding of healing modes and a genuine cooperation of surgeons, scientists, and engineers in order to stop reinventing the same things. 

## 5. Conclusions

Complete endothelialization of cardiovascular grafts enabling an undisturbed blood flow remains a significant challenge in the areas of cardiovascular surgery and regenerative therapies. Despite extensive research, no bioartificial implant capable of fully restoring cardiovascular functions or replacing native tissues has been identified. One of the remaining challenges is to mimic the function of the EBM given the complexity of its composition and molecular architecture. Among the methods being explored in fundamental research, approaches based on EBM-derived biopolymers or cell-derived matrices seem promising. A directed three-dimensional arrangement of ECM fibers with a defined spacing of integrin binding sites to support EC adhesion might be beneficial, as shown for self-assembled peptide hydrogels [126]. However, one should bear in mind that such constructs should be manufactured in line with the motto “the simpler, the better” to facilitate the standardization of the production process with regard to quality requirements necessary for clinical translation. Keeping the number of initial materials and processing steps low as well as avoiding concepts comprising preseeding of cells on the device before implantation might finally leverage the use of this technology in clinical practice.

## Data Availability

Not applicable.
